# LncRNAs and Rheumatoid Arthritis: From Identifying Mechanisms to Clinical Investigation

**DOI:** 10.3389/fimmu.2021.807738

**Published:** 2022-01-11

**Authors:** Wentao Huang, Xue Li, Chen Huang, Yukuan Tang, Quan Zhou, Wenli Chen

**Affiliations:** ^1^ Ministry of Education (MOE) Key Laboratory of Laser Life Science and Institute of Laser Life Science, College of Biophotonics, South China Normal University, Guangzhou, China; ^2^ Guangdong Provincial Key Laboratory of Laser Life Science, College of Biophotonics, South China Normal University, Guangzhou, China; ^3^ Guangzhou Key Laboratory of Spectral Analysis and Functional Probes, College of Biophotonics, South China Normal University, Guangzhou, China; ^4^ Department of Minimally Invasive Interventional Radiology, Guangzhou Panyu Central, Hospital, Guangzhou, China; ^5^ Department of Radiology, The Third Affiliated Hospital of Southern Medical University, Guangzhou, China

**Keywords:** LncRNAs, rheumatoid arthritis, pathological features, diagnosis, therapy

## Abstract

Rheumatoid arthritis (RA) is a systemic chronic autoinflammatory disease, and the synovial hyperplasia, pannus formation, articular cartilage damage and bone matrix destruction caused by immune system abnormalities are the main features of RA. The use of Disease Modifying Anti-Rheumatic Drugs (DMARDs) has achieved great advances in the therapy of RA. Yet there are still patients facing the problem of poor response to drug therapy or drug intolerance. Current therapy methods can only moderate RA progress, but cannot stop or reverse the damage it has caused. Recent studies have reported that there are a variety of long non-coding RNAs (LncRNAs) that have been implicated in mediating many aspects of RA. Understanding the mechanism of LncRNAs in RA is therefore critical for the development of new therapy strategies and prevention strategies. In this review, we systematically elucidate the biological roles and mechanisms of action of LncRNAs and their mechanisms of action in RA. Additionally, we also highlight the potential value of LncRNAs in the clinical diagnosis and therapy of RA.

## 1 Introduction

**Figure 1:**
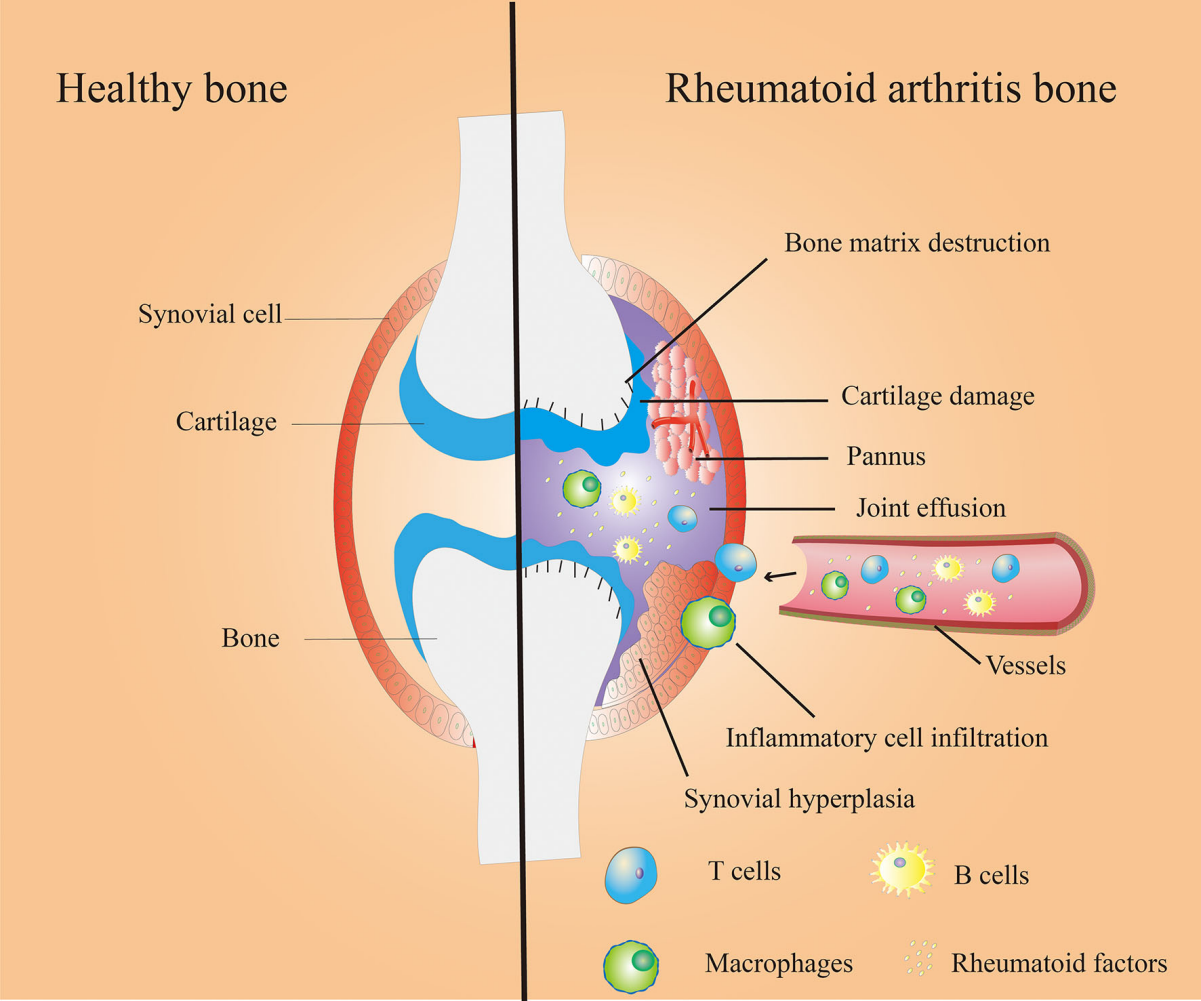
Bone microenvironment in healthy (left) and RA (right) bone. Healthy bones have thin and smooth synovium, smooth cartilage tissue and healthy bone matrix (left); Synovial hyperplasia, pannus formation, inflammatory cell infiltration, cartilage damage, bone matrix destruction, joint effusion and other symptoms in Rheumatoid arthritis (RA) bone (right).

Autoimmune diseases cause tissue damage through the physiological immune response to autoantigens. Rheumatoid arthritis (RA) is a systemic autoimmune disorder caused by autoimmune response in inflammatory synovial tissue and subsequent joint damage. The degree of joints injury of RA gradually intensified overtime, and eventually led to varying degrees of joint dysfunction and deformities (1). **Figure 1** shows the bone microenvironment of healthy bone and RA bone. RA is a systemic disease with frequent involvement of other organs besides the joints. The common comorbidities are RA–Interstitial Lung Disease (2), Uveitis (3), Sjögrens syndrome (4) etc.

As of now, RA cannot be completely cured, and current clinical drugs can only relieve pain or reduce the development of inflammation (5). Disease Modifying Anti-Rheumatic Drugs (DMARDs) are the therapy of choice for RA. This method treats RA with methotrexate (MTX) alone or in combination with other drugs. However, the latest data shows that therapy is nearly ineffective in about 40% of RA patients. Worse, it can put them at risk of MTX-related side effects (6). It is therefore necessary to summarize the current research advances and mechanisms of RA, which could be very useful in discovering a more effective and precise therapy.

**Figure 2:**
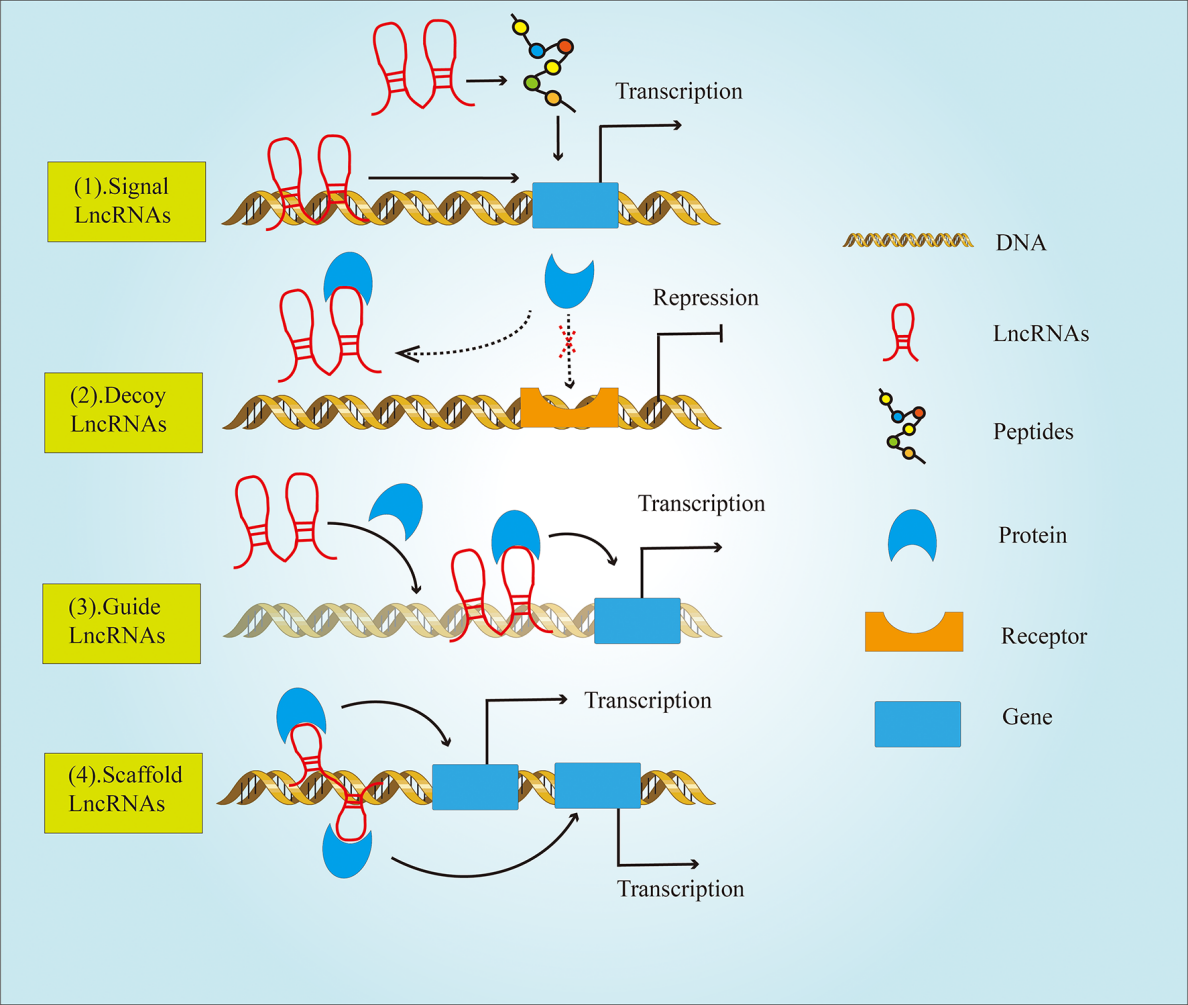
The classification of LncRNAs based on their functions. LncRNAs are divided into four types: (1) Signal LncRNAs; (2) Decoy LncRNAs; (3) Guide LncRNAs; (4) Scaffold LncRNAs.

It is well established that the genomes of most species can produce a large amount of LncRNAs during the transcription process (7). Originally, LncRNAs were believed to be a byproduct of RNA polymerase II transcription that had no biological function. However, Borsani etal. (8) discovered LncRNA Xist, which could regulate the inactivation of the X chromosome. Since then, the function of LncRNA has attracted wide attention from scientists. Functionally, LncRNAs can be divided into four major classes (**Figure 2**) (9), including: (1) Signal LncRNAs can be used as molecular signals to quickly perform their potential regulatory functions without protein translation. (2) Decoy LncRNAs, acting as molecular sponges, bind with transcription factors and sequesters them from target genes (3) Guide LncRNAs bind ribonucleoprotein complexes, which are localized to specific DNA sequence (4) Scaffold LncRNAs play the role of a central platform. This type of LncRNAs can realize the exchange of information and the integration between different signaling pathways by combining with several related transcription factors.

LncRNAs play an important role in various biological processes and are related to the pathogenesis of different kinds of diseases. Growing evidence links LncRNAs with the risk of multiple immune diseases such as RA, Systemic Lupus Erythematosus (10), etc. In fact, as the important regulator of immune cell differentiation and activation, LncRNAs have been generally recognized for its role in autoimmune processes and autoimmune diseases (11). There are a number of factors responsible for the disfunction of LncRNAs in RA. One of the major reasons is the enrichment of inflammatory cytokines in RA. There was evidence that tumor necrosis factor-α (TNF-α) treatment raised the level of H19 in fibroblast-like synovial cells (FLSs) (12). Meanwhile, TNF-α treatment could significantly inhibit the expression level of LncRNA PINT in FLSs (13). It has also been reported that the promoter of LncRNA MEG3 was significantly methylated in an inflammatory environment (14). In addition, the dysregulation of LncRNAs expression in RA may also be caused by bacterial infection. The bacterial cell wall components are lipopolysaccharide (LPS) and peptidoglycan (PGN). LPS and PGN could enhance the expression of lncRNA HIX003209, which reversely promoted the proliferation and activation of macrophages through inhibitor of nuclear factor κB (NF-κB) alpha (IκBα)/NF-κB signaling pathway (15).

In this article, we will focus on the overview of the biological functions of LncRNAs in RA and their regulatory mechanism on the process of RA. Moreover, we will also discuss the prospects of LncRNAs in the clinical diagnosis and therapy of RA.

## 2 LncRNAs and Immune Microenvironment

RA is an irreversible chronic autoimmune disease. Autoimmune diseases have both prominent inflammatory responses and dysfunctional immune responses (16).

RA is accompanied by excessive production of inflammatory cytokines (including interleukin-1-β (IL-1β)and TNF-α, etc.) (17). RA synovial tissue contains T cells, B cells, and macrophages, which reflects an active local immune response (18). The most recent studies suggest that some LncRNAs are associated with prominent inflammatory responses and dysfunctional immune responses.

### 2.1 LncRNAs Modulate the Release of Inflammatory Cytokines

**Figure 3:**
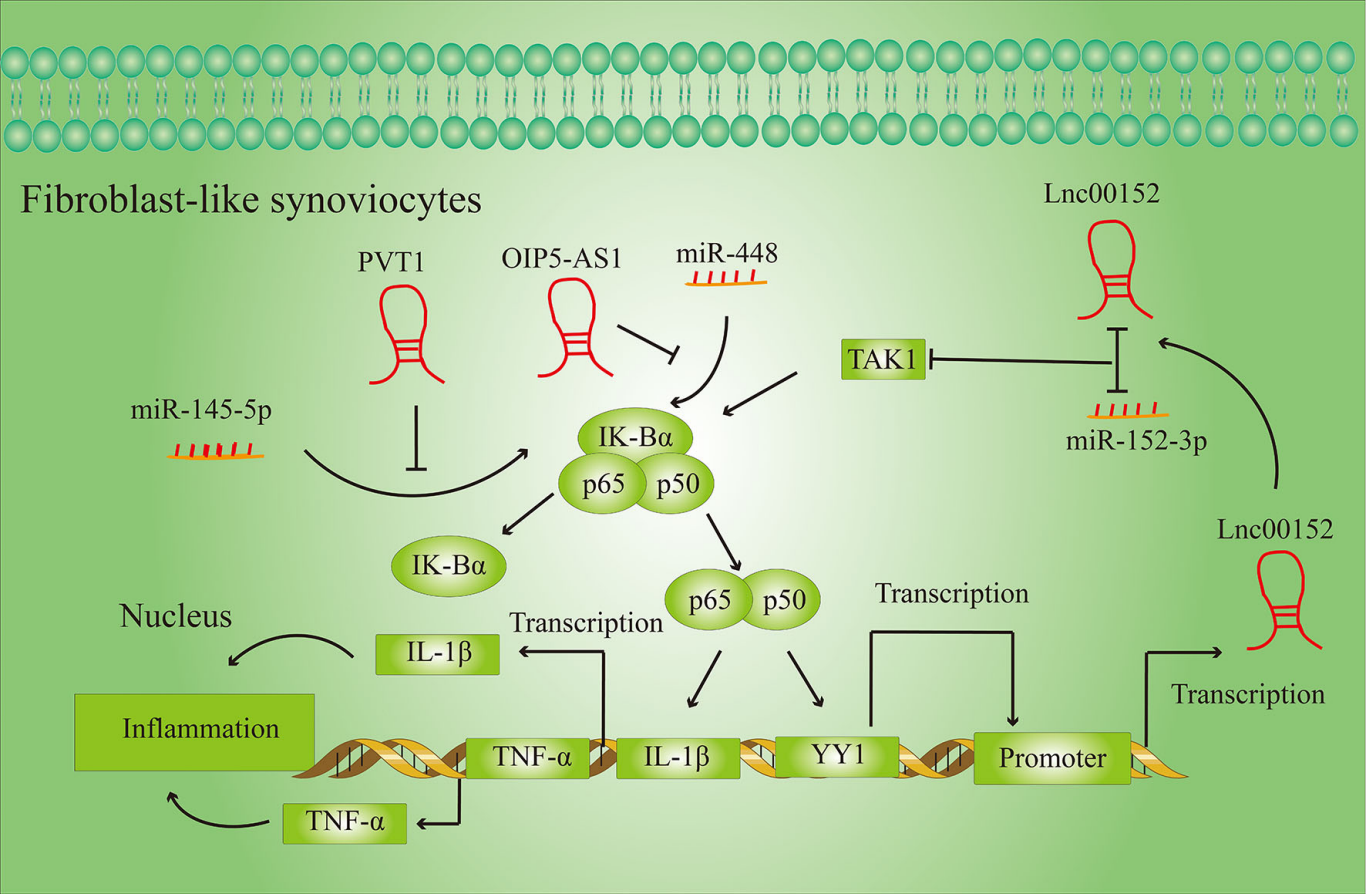
LncRNAs regulate the secretion of inflammatory cytokines in FLSs. LncRNAs regulate the secretion of inflammatory cytokines by activating or inhibiting specific signaling pathways in fibroblast-like synovial cells (FLSs).

Inflammation is a complex anti-disease response that reflects the body’s response to various harmful stimuli (19). Inflammatory cytokines are mediators of inflammatory and immune system responses. Excessive pro-inflammatory factors in the body can exacerbate cell necrosis and tissue degeneration, and lead to a variety of diseases. LncRNAs can play a negative or positive role in the secretion of inflammatory factors through various molecular mechanisms (**Figure 3**).

Inflammation affects all stages of RA development, and LncRNAs can regulate the release of inflammatory cytokines in RA. For example, Lnc00152, which is upregulated in RA, activates the TGF-β-activated kinase 1 (TAK1)-mediated NF-κB signaling pathway by targeting miR-103a (20). Activation of the NF-κB signaling pathway can activate the transcription of inflammatory factors such as TNF-α and IL-1β, thereby intensify the inflammatory response in the tissue. At the same time, the NF-B signaling pathway can also increase the transcription activity of the transcription factor YY1, which by binding to the promoter of Lnc00152 directly promotes the expression of Lnc00152.

PVT1 and OIP-AS5 also regulate the expression of pro-inflammatory factors by regulating the NF-κB signaling pathway. PVT1 regulates NF-κB signaling by targeting miR-145-5p (21), while OIP-AS5 influences expression of NF-κB signaling pathway by targeting miR-448 (22).

Some studies have only shown that LncRNAs could regulate the expression of pro-inflammatory factors in RA patients, but the specific regulatory mechanism is still unclear. For example, Ma et al. (23) found that, compared to healthy controls, the expression of LncRNA GAS5 in the plasma of RA patients was significantly downregulated, while interleukin-18 (IL-18) was upregulated, and there was a significantly negative correlation between LncRNA GAS5 and IL-18. This only proves that LncRNA GAS5 is correlated with the expression of pro-inflammatory factors. A study by Tao et al. (24) proved that the downregulation of GAS5 in RA patients is an important reason for the upregulation of the proinflammatory factor TNF. The overexpression of GAS5 can significantly hinder the expression of TNF in FLS.

Taken together, this evidence shows that LncRNAs play an important role in regulating body’s inflammatory response. Therefore, reducing the release of inflammatory factors though targeted LncRNAs may offer a new approach to RA therapy.

### 2.2 LncRNAs Modulate T Cell Differentiation

**Figure 4:**
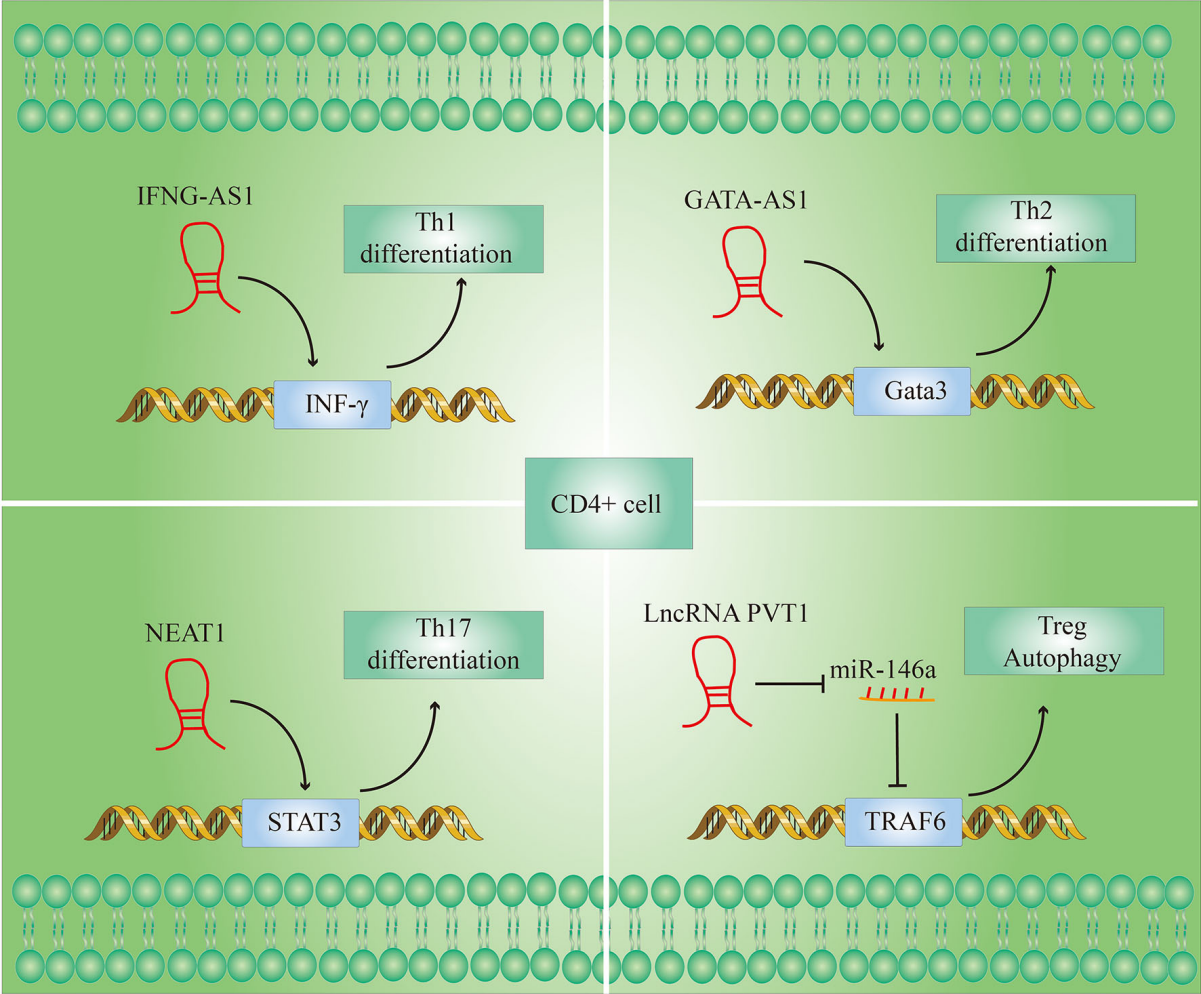
LncRNAs modulate T cell differentiation. (1) IFNG-AS1 affects the transcription of interferon γ (IFN-γ) genes during Th1 differentiation; (2) Gata3-AS1 enhances Th2 cell differentiation by enhancing Gata3 transcription; (3) NEAT1 promotes the differentiation of CD4+ T cells into Th17 cells by increasing the level of signal transducer and activator of transcription 3 (STAT3) protein; (4) lncRNA PVT1 promotes autophagy in Treg cells by targeting miR-146a.

The autoreactive T cell (CD4+ T cell) is crucial for the pathogenesis of RA. CD4 + T cells are composed of several subsets, including helper T (Th) 1, Th2, Th17, and regulatory T (Treg) cells (25). **Figure 4** shows the role of LncRNAs in the process of T cell differentiation.

An imbalance of Th1/Th2 cell ratio in peripheral blood may play an important role in the pathogenesis of RA. The Th1/Th2 cell ratio of RA patients is significantly higher than that of healthy individuals, and a reduction in the ratio has a therapeutic effect on RA (26). An increase in the Th1/Th2 ratio can cause an increase in interferon γ (IFN-γ) produced by Th1 and a decrease in Interleukin-4 (IL-4) produced by Th2, leading to increased inflammation (27).

LncRNA IFNG-AS1 influences the transcription of IFN-γ-encoding genes during Th1 differentiation (28). The transcript levels of IFNG-AS1 in PBMC (Peripheral blood mononuclear cell) of RA patients were significantly higher than in healthy controls (29). And their previous studies have shown that the knockdown of IFNG-AS1 would lead to a significant decrease in IFNG transcript levels and the proportion of Th1 cells there are differentiated from CD4 + T cells *in vitro* (30).

Not only Th1 cells but also Th2 cells function activation is also regulated by LncRNAs. LncRNA GATA-AS1 is required for efficient transcription of the transcription factor GATA3 gene, and GATA3 is considered to be the main transcriptional regulator of Th2 lineage commitment (31). GATA3-AS1 thus potentiates Th2 cell differentiation by enhancing GATA3 transcription (32).

Apart from that, T17 cells and Treg cells also may play a role in the pathogenesis of RA. T17 cells have been shown to promote inflammation and their function is known to be over-activated in the peripheral blood of RA patients. In contrast to T17 cells, although Treg cells are also derived from CD4 + T cells, but they have an anti-inflammatory effect in RA. The immune balance of Treg and Th17 cells is very important in maintaining the normal immune status of the body, and this imbalance is one of the major reasons for the occurrence of RA (33). During the rapid development of RA, Th17 cells in the patients’ peripheral blood increased while Treg cells decreased, and there was an apparent imbalance in the immune (34).

Given the importance of the Th17/Treg immune imbalance in the course of RA, restoring Th17/Treg immune balance may be an important method for RA therapy. Shui et al. (35) found that LncRNA NEAT1 was significantly upregulated in the PBMC of RA patients. When the differentiation of CD4 + T cells to Th17 cells was induced *in vitro*, the expression of LncRNA NEAT1 increased significantly, and knockdown of NEAT1 can significantly inhibit the differentiation of CD4 + T cells to Th17 cells. Further investigations showed that the signal converter and activator of transcription 3 (STAT3) is a key molecule for the differentiation of CD4 + T cells into Th17 cells and is also a downstream molecule of NEAT1. NEAT1 in cells may play a role in regulating cell function by influencing the level of expression of STAT3. In summary, these studies indicate that the knockdown of NEAT1 inhibits the differentiation of CD4 + T cells into Th17 cells by reducing STAT3 levels and thereby having an anti-inflammatory effect.

There are currently only a few studies on LncRNAs that regulate the proportion of Treg cells and the levels of Treg-related molecules in RA patients. However, there have been many related reports in other disease models. For example, LncRNA PVT1 regulates the expression of TNF receptor-associated factor (TRAF) 6 in Treg cells by targeting miR-146a to promote autophagy of Treg cells. Increased autophagy promoted Tregs to suppress the expression of the Th17 signature factor interleukin-17 (IL-17), whereby the Th17 expression was reduced (36). After overexpression of LncRNA PVT1, the number of Th17 cells decreased, the Treg cells increased and the Treg/Th17 ratio increased in spinal cord and spleen cells cultured *in vitro* (37). In addition, the researchers found that the LncRNA RP11-340F14.6 could stimulate Th17 differentiation and inhibit Treg proliferation in juvenile idiopathic arthritis (38).

In conclusion, these evidences indicate that LncRNAs indeed play a role in regulating the progression of RA by regulating the balance of Th1/Th2 and Th17/Treg ratios in peripheral blood. Therefore, research into the mechanism of LncRNAs and T cell activation can provide new ideas for the therapy of RA.

### 2.3 LncRNAs Modulate B Cells Rheumatoid Factor (RF) Secretion

Although T cell activation is viewed as a key component in the pathogenesis of RA, there has long been evidence that this activation is influenced by B cells (39). Therefore, B cells are also an essential factor in the pathogenesis of RA. As we all know, the synovium of patients with RA contains large numbers of plasma cells that can produce RF. The plasma cells are differentiated from B cells. Studies have shown that RF-positive is associated with more aggressive joint disease, higher frequency of extra-articular manifestations, and higher mortality (40). Recent evidence suggests that the LncRNAs play an important role in regulating RF secretion.

According to previous reports, some LncRNAs were positively correlated with RF levels in RA. The expression of LncRNA ENST00000619282 in the PBMC of RA patients was significantly increased. The expression of ENST0000061928 in the PBMC of RA patients correlates positively with the expression of RF. The down-regulation of the LncRNA ENST0000061928 can reduce the expression of RF in RA (41). Similarly, LncRNA HIX003209 has also been reported to be upregulated in RA and positively correlated with RF levels (15).

In addition, some LncRNAs correlated negatively with RF levels in RA. For example, it was found that the expression of RF was significantly increased in RA patients, while the expression of Lnc00638 was significantly reduced by comparison with healthy controls in clinical samples. Association rule analysis showed that the decrease in Lnc00638 expression was significantly related to the increase in RF levels (42). Obviously, the interaction between LncRNAs and B cells is an important part of the development of RA, but there have not been many studies on the mechanism of LncRNAs and B cells in RA. Therefore, further research on LncRNAs in B cells may provide new insights for the therapy of RA.

### 2.4 LncRNAs Modulate the Differentiation of Macrophages

In general, macrophages are divided into 2 types: the M1 macrophage phenotype secretes pro-inflammatory factors and causes tissue destruction, while the M2 macrophage phenotype secretes anti-inflammatory factors and controls tissue regeneration (43). The inflammatory immune response in the body of RA patients directly affects the polarization of macrophages in the peripheral blood, synovium and synovial fluid, which increases the number of pro-inflammatory macrophages of the M1 type, which disrupts the M1/​​M2 balance (44). Recently, an increasing number of studies have found that LncRNAs influence the progression of RA by regulating the polarization of macrophages.

LncRNA H19 was examined in mouse models with RA and adjuvant-induced arthritis and found to be upregulated (45). Treating macrophages with IFN-γ and LPS can induce their polarization to M1 (46), while silencing LncRNA H19 can inhibit this polarization. At the same time, the expression of some pro-inflammatory genes Interleukin 6 (IL-6), CD80, chemokine (C-C motif) ligand 8 (CCL8) and C-X-C motif chemokine 10 (CXCL10) in macrophages can also decrease with the silencing of LncRNA H19. LncRNA HIX003209 was also significantly increased in RA patients. HIX003209 activates the IκBα/NF-κB signaling pathway by targeting miR-6089 in macrophages, which can promote macrophage proliferation and M1 polarization (15).

Therefore, reducing M1 type macrophages and increasing M2 type macrophages can restore the dynamic balance of M1/M2 by targeting LncRNAs associated with macrophage polarization, which is expected to become a new strategy for RA treatment.

## 3 The Relationship Between Lncrnas And The Pathological Features of RA

The main pathological features of RA include synovial hyperplasia, pannus formation, and articular cartilage damage, as well as bone matrix destruction. As shown in **Figure 1**. There is growing evidence that LncRNAs are involved in the course of RA. The following is a summary of the current functional mechanisms of LncRNAs involved in RA progression.

### 3.1 ncRNAs Affect Synovial Hyperplasia

**Figure 5:**
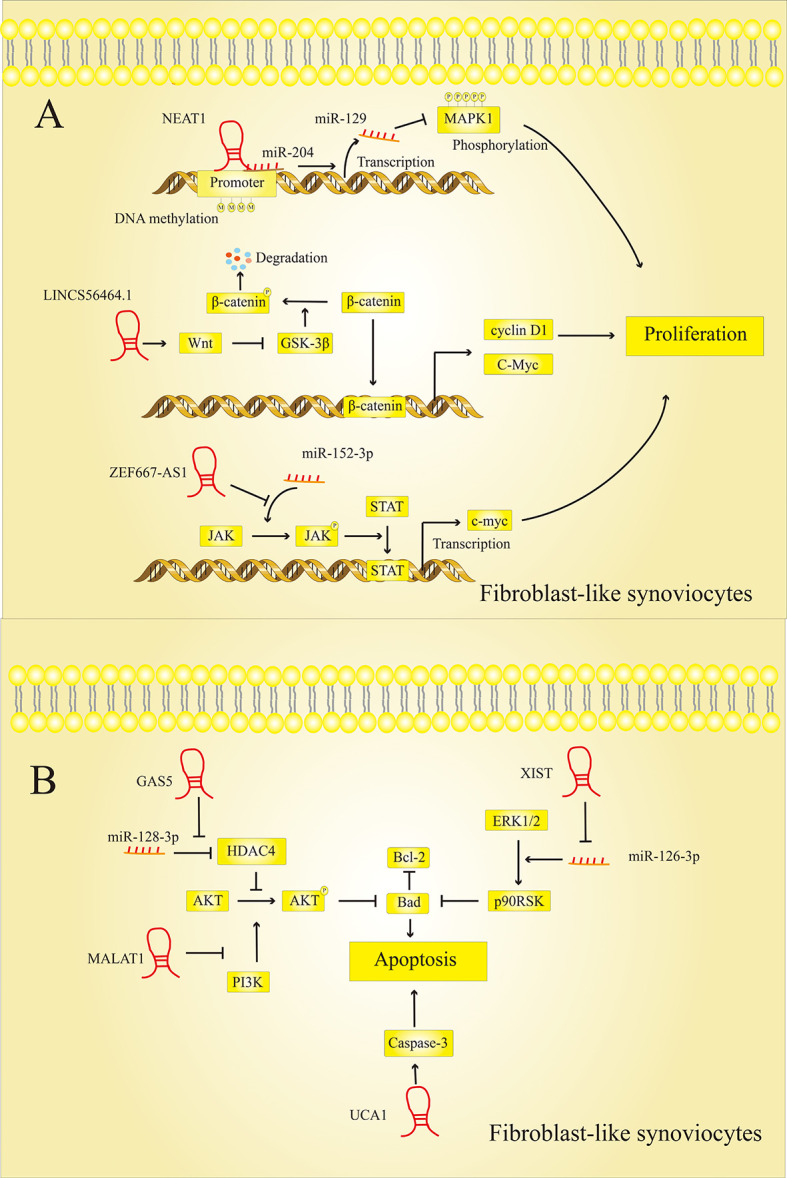
LncRNAs modulate FLSs cell proliferation and apoptosis. **(A)** LncRNAs regulate the proliferation of FLSs: LncRNAs can regulate the expression of MAPK1, c-myc, cyclin D1 and other proteins to affect the proliferation of FLSs. **(B)** LncRNAs regulate the apoptosis of FLSs: LncRNAs can regulate the expression of Bad, Bcl-2, caspase-3 and other proteins to affect the proliferation of FLSs.

The synovial membrane is a thin membrane that covers the inner surface of the fibrous joint capsule, tendon sheath, and bursa (47). The synovial membrane of the joint is a dynamic environment in which the synovial fluid secreted by the synovial membrane can lubricate the joint space. At the same time, the synovial fluid is constantly circulated and updated when the joints are active in order to maintain normal joint function (48). Synovial hyperplasia can lead to an increase in synovial fluid and at the same time the reabsorption of synovial fluid though the synovial membrane also be affected. Clinical signs of synovial hyperplasia were joint swelling or effusion (49). Synovial hyperplasia is mainly caused by the excessive proliferation of FLSs (50) and the disruption of FLSs apoptosis (24). Recent studies have shown that LncRNAs affect synovial hyperplasia by activating or inhibiting signaling pathways related to proliferation and apoptosis in FLSs. **Figure 5** summarizes some of the pathways in which LncRNAs participate in the proliferation or apoptosis of FLSs.

#### 3.1.1 LncRNAs Regulate FLSs Cell Proliferation in RA Lesions

LncRNAs regulate the proliferation of FLSs in RA lesions by activating or inhibiting specific signaling pathways. However, the mechanisms by which these LncRNAs affect synovial cell proliferation are not entirely consistent.

Chen et al. (51) found that LncRNA NEAT1, which is highly expressed in RA, could bind to miR-204, thereby promoting the methylation of the miR-129 promoter. Extracellular regulated protein kinases (ERK1/2), which are closely related to cell proliferation and differentiation, are the target genes of miR-129. The increased expression of miR-129 in RA inhibits the phosphorylation of ERK1/2 and promotes the proliferation of FLSs. LncRNA-S56464.1, which activates the downstream wingless and int1 (Wnt) signaling pathway and can promote the proliferation of synovial cells, is also strongly expressed in the lesions of RA patients (52). In general, the activity of glycogen synthase kinase 3 beta (GSK-3) promotes the phosphorylation of catenin and its breakdown (53). LncRNAS56464.1 can inhibit GSK-3 *via* the Wnt pathway, which means that more β-catenin can penetrate the cell nucleus and exercise its function as a transcription factor. Once β-catenin has reached the nucleus, it can initiate the expression of genes that promote cell division, such as cellular myelocytomatosis oncogene (c-myc) and cyclin D1, which promotes cell proliferation. In contrast, the LncRNA ZNF667-AS1 was significantly downregulated in RA tissues and FLSs compared to normal tissues and cells (54). LncRNA ZNF667-AS1 acts as a competitive sponge for miR-523-3p in RA lesions and thereby inhibits the activation of miR-523-3p on the Janus kinase (JAK)/STAT signaling pathway. Activation of the JAK/STAT signaling pathway can induce c-myc transcription, which is related to cell proliferation (55). Therefore, when the expression of LncRNA ZNF667-AS1 is inhibited, the expression of downstream c-myc increases, thereby promoting FLSs proliferation and exacerbating the condition of RA.

#### 3.1.2 LncRNAs Regulate FLSs Cell Apoptosis

LncRNAs can also regulate the apoptosis of FLSs in RA patients by influencing specific signaling pathways.

It seems that some LncRNAs promote apoptosis of the FLS and thereby suppress RA progression. Previous studies have shown that LncRNA GAS5 was downregulated in RA (24). As a competitive sponge for miR-128-3p, GAS5 can upregulate the expression of histone deacetylase-4 (HDAC4). After GAS5 has been downregulated in RA, the expression of HDAC4 in the cell is inhibited, thereby increasing the phosphorylation level of protein kinase B (AKT) in the AKT/mammalian target of rapamycin (mTOR) signaling pathway. In addition, there is already evidence that activated Akt Bad, a member of the pro-apoptotic B-cell lymphoma-2 (Bcl-2) family, phosphorylates and induces its inactivation (56). Therefore, downregulation of LncRNA GAS5 can promote cell apoptosis. The expression of LncRNA UCA1 was suppressed in RA synovial tissue (57). The researchers found that depressing the expression level of UCA1 may reduce the expression level of apoptosis-related cysteinyl aspartate specific proteinase 3 (caspase-3) to inhibit cell apoptosis and promote the progression of RA.

At the same time, studies have shown that some LncRNAs can promote the progression of RA by inhibiting the apoptosis of the FLSs. The researchers analyzed the association between LncRNA DC, ANRIL, MALAT1, ZFAS1 levels and RA patients using qRT-PCR. The results indicate that LncRNA DC, ANRIL, MALAT1, ZFAS1 may be involved in the occurrence and development of RA (58). Among them, it was found that LncRNA ZFAS1 is strongly expressed in the synovial tissue of RA. A disintegrin and metalloproteinase with thrombospondin motifs 9 (ADAMTS9) is a metalloproteinase and its down-regulation is associated with decreased cell proliferation and increased cell apoptosis (59). LncRNA ZFAS1 inhibits the apoptosis of fibroblast-like synoviocytes *via* the miR-2682-5p/ADAMTS9 axis in RA (60). In addition, Liu et al. (61) found that LncRNA XIST is also highly expressed in synovial tissues. The target relationship between miR-126-3p and XIST was confirmed by a dual luciferase reporter gene assay. XIST can promote the expression of Bcl-2, a protein related to cell apoptosis, by targeting miR-126-3p, thereby reducing the rate of cell apoptosis. And some studies have shown that miR-126-3p inhibits the expression of Bcl-2 *via* Erk1/2-related signaling pathways (62). This indicates that XIST inhibits the rate of apoptosis in RA through the XIST/miR-126-3p/Erk1/2 axis.

The excessive proliferation of FLSs and the dysregulation of the apoptotic pathway are important reasons for synovial hyperplasia and the development of RA. Therefore, the inhibition of cell proliferation and the induction of apoptosis are necessary for the effective therapy of RA. Targeting LncRNAs to promote proliferation and inhibit apoptosis in FLSs of RA may provide a new method for RA therapy.

### 3.2 LncRNAs Regulates Pannus Formation

As RA symptoms continue to develop, new pathological features appear in the joints: pannus. Pannus is a fibrous structure made up of numerous neovascularizations, hypertrophic synovial cells, and inflammatory cells (63). It secretes inflammatory cytokines and proteolytic enzymes and causes joint disease. And it behaves like an aggressive neoplasm that expands into the joint space and adheres to the cartilage surface, preventing the bones from absorbing nutrients from the synovial fluid (64). Angiogenesis is an important event in the formation and maintenance of pannus in RA (65). At the same time, another key factor in pannus formation is that FLSs show invasive behavior (66). Several recent studies have implicated LncRNAs as an essential molecule in pannus formation.

#### 3.2.1 LncRNAs Regulate Angiogenesis

Some pro-angiogenic factors such as vascular endothelial growth factor (VEGF) and TNF-α, etc. can stimulate vasculogenesis. LncRNA has been shown to regulate the process of angiogenesis by regulating the release of pro-angiogenic factors (67).

Clinically, the expression of LncRNA MEG3 is downregulated in RA patients. Knockdown of the expression of LncRNA MEG3 can significantly increase the expression of VEGF. This suggests that the knockdown of LncRNA MEG3 can promote angiogenesis (68). Similarly, ZNF667-AS1 was downregulated in LPS stimulated chondrocytes. The inhibition of the expression of ZNF667-AS1 can significantly promote the expression of TNF- in chondrocytes and thereby increase the angiogenic activity (54).

#### 3.2.2 LncRNAs Regulate the Migration and Invasion Ability of FLSs

**Figure 6:**
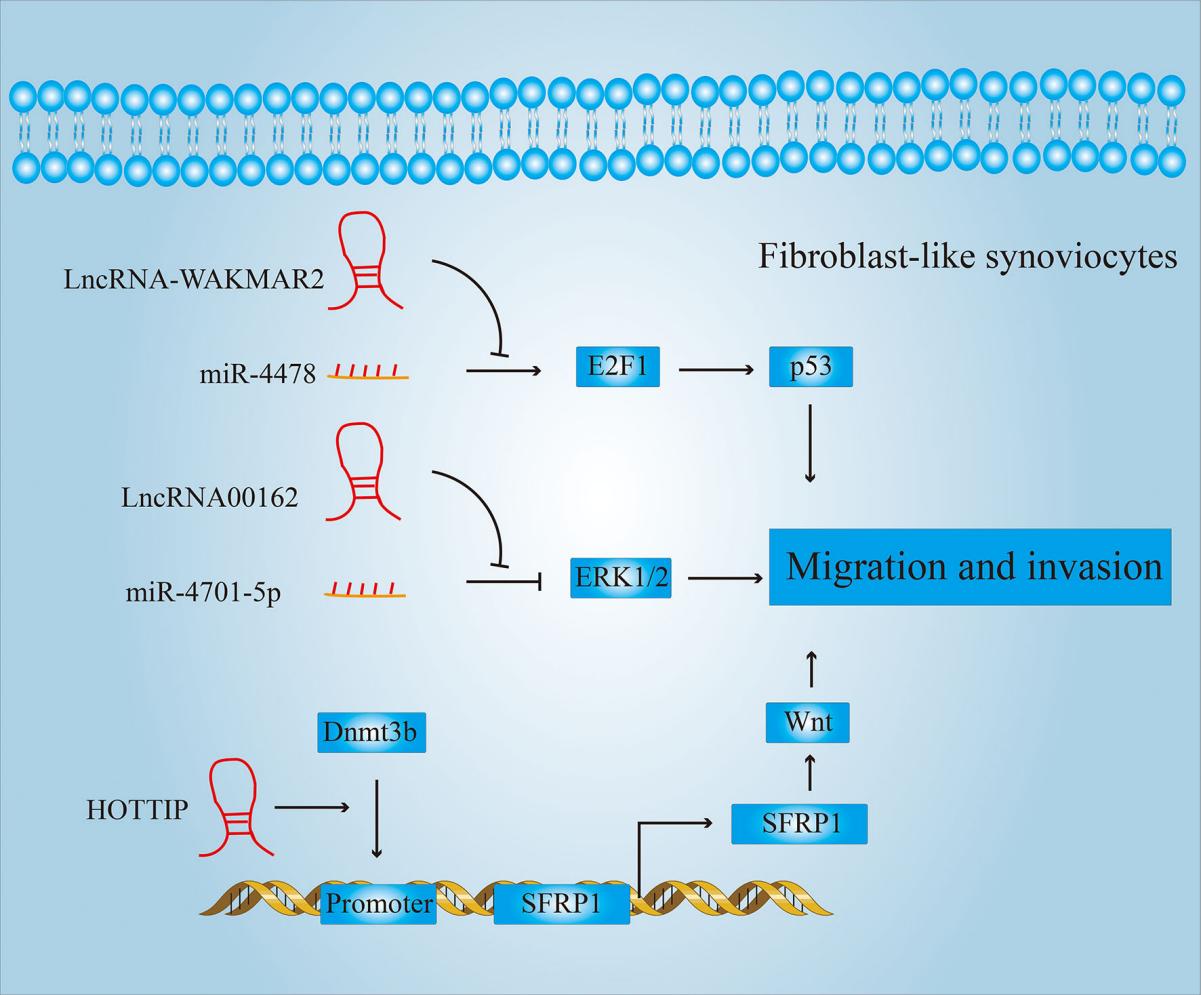
LncRNAs regulate the migration and invasion of FLSs. LncRNAs can regulate the expression of p53, p38, wnt and other proteins, thereby affecting the migration and invasion of FLSs.

Many drugs currently treat RA by blocking the migration and invasion-related signaling pathways of FLS. **Figure 6** summarizes some of the pathways in which LncRNAs participate in the migration or invasion of FLSs.

For example, drugs used in clinical therapy of RA, such as (5R)-5-hydroxy triptolide (LLDT-8), etc., have been found to have a good therapeutic effect by inhibiting the migration and invasion of FLSs (69). Zhou et al. also found that after treatment with LLDT-8, the expression of LncRNA WAKMAR2 was significantly higher than that of the untreated group. Further studies have shown that WAKMAR2, as a competitive sponge of miR-4478, inhibits the downstream E2F1 (E2F transcription factor 1)/p53 signaling pathway and thereby reduces the migration and invasion properties of FLSs. LINC00162 also acts as a competitive sponge for miR-4701-5p and promotes migration and invasion of FLSs. The knockdown of LINC00162 inhibits the migration and invasion of FLSs by suppressing ERK1/2 activity (50).

In addition, LncRNAs can also promote activation of signaling pathways related to migration and invasion by inducing methylation of the promoters of key genes. For example, HOTTIP can induce the recruitment of DNA (Cytosine-5-)-Methyltransferase 3 Beta (DNMT3B) to the Secreted Frizzled Related Protein 1 (SFRP1) promoter (70). Activated SFRP1 can activate the Wnt signaling pathway and improve the ability of FLSs to migrate and invade, which can promote the development of pannus.

### 3.3 LncRNAs Regulate Articular Cartilage Damage and Bone Matrix Destruction

Osteoclasts are a type of pluripotent stem cells that originate in bone marrow and are known for bone resorption. Articular cartilage, the connective tissue made up of cartilage cells, can reduce friction in the joints and act as a shock absorber in the joints (71). RA leads to secondary joint cartilage damage and destruction of the bone matrix, which leads to joint deformities (72).

In general, osteoclast differentiation, usually controlled by receptor activator of nuclear factor-κ B ligand (RANKL), can be enhanced under the inflammatory conditions of RA (73). After excessive proliferation and differentiation of osteoclasts, excessive bone erosion leads to cartilage damage and bone matrix destruction.

The researchers found significantly lower Hotair levels in differentiated osteoclasts and rheumatoid synoviocytes. After introducing a lentiviral construct containing Hotair into osteoclasts and synovial cells to upregulate Hotair, the activities of matrix metalloproteinase-2 and matrix metalloproteinase-13 were significantly reduced (74). This can moderate the breakdown of the bone and cartilage matrix, thereby reducing joint damage.

**Figure 7:**
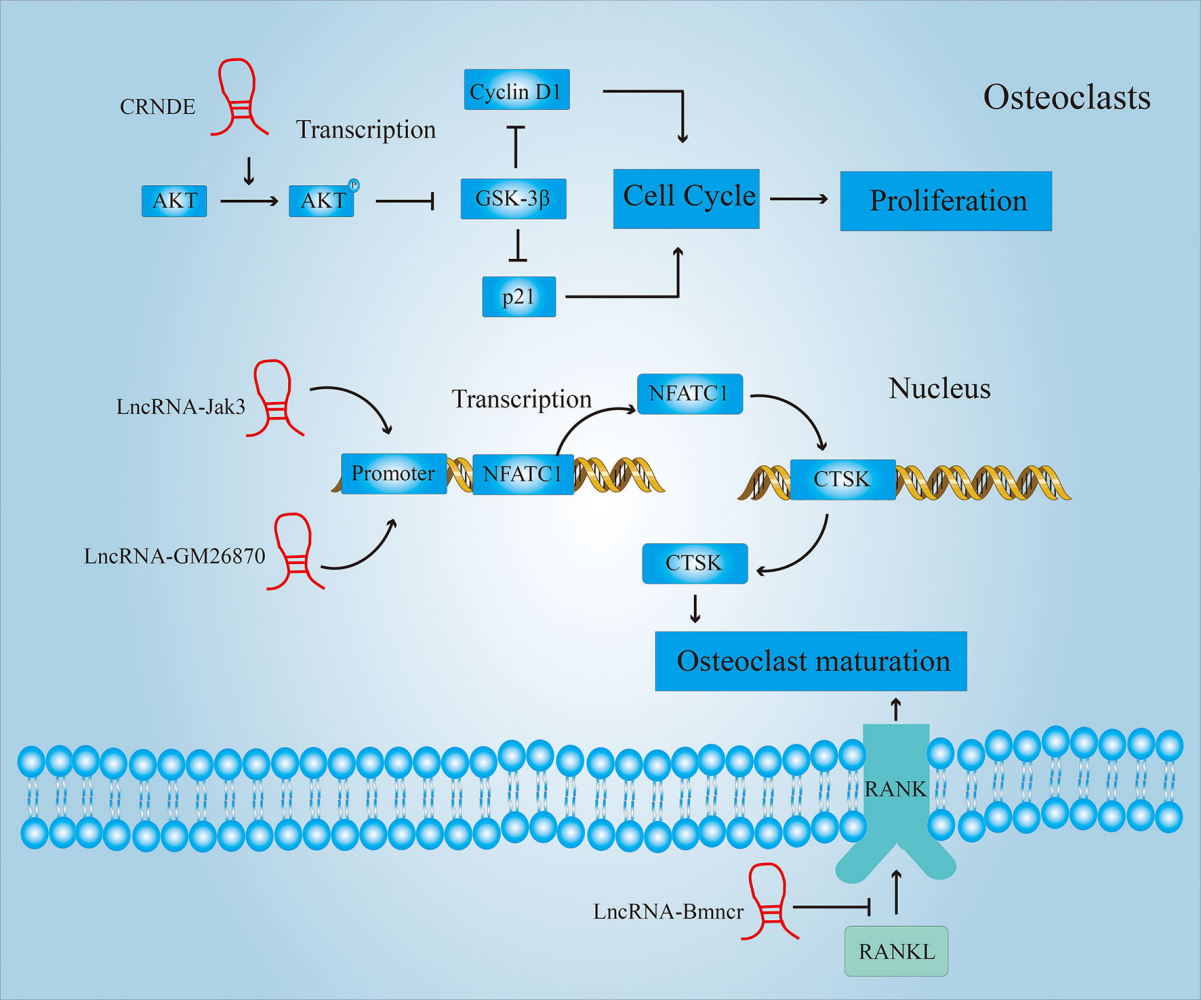
LncRNAs regulate the proliferation and maturation of osteoclasts. LncRNAs regulate the proliferation of osteoclasts by affecting the cell cycle; LncRNAs regulate the maturation of osteoclasts by affecting the expression of Cathepsin K (CTSK)and receptor activator of nuclear factor-κ B (RANK).

There are many reports showing that LncRNA can promote proliferation and maturation of osteoclasts in other disease models. **Figure 7** shows some of the ways in which LncRNAs participate in the proliferation or maturation of osteoclasts. This provides us with a basis for predicting the role LncRNA can play in RA. For example, CRNDE is a newly discovered LncRNA with critical role in osteoclastogenesis and bone resorption. Studies have shown that the phosphatidylinositol 3-kinase (PI3K)/AKT signaling pathway plays a significant role in the regulation of osteoclast proliferation, differentiation and apoptosis (75). Li et al. found that CRNDE inhibits the expression of GSK-3β by promoting the phosphorylation of AKT (76). And GSK3-β can inhibit cell proliferation by inhibiting the expression of cell cycle-related proteins cyclin D1 and p21. Therefore, over-expression of CRNDE can reverse the GSK3-mediated inhibition of osteoclast growth.

Cathepsin K (CTSK) is a lysosomal cysteine ​​protease that is involved in bone resorption (77). It plays a central role in bone resorption and is a sign of osteoclast maturation. Lee et al. (78) found that the activation of nuclear factor of activated T cells 1 (NFATC1) mediated by LncRNA Jak3 upregulated the expression of CTSK. And the down-regulation of the expression of LncRNA Jak3 inhibits the formation of mature osteoclasts. LncRNA GM26870 also promotes osteoclast maturation by mediating the activation of NFATC1 (79).

In addition to up-regulating the expression of CTSK, activating the RANKL-RANK (receptor activator of nuclear factor-κ B) signaling pathway is also an important means to promote the development and maturation of osteoclasts (80). Chen et al. (81) found that the expression of LncRNA Bmncr was significantly lower in osteoporotic mice compared to normal mice. Through further research, it was found that LncRNA Bmncr can inhibit osteoclast differentiation by inhibiting the RANKL-RANK signaling pathway.

Articular cartilage damage is an important pathological feature of rheumatoid arthritis, and it is also very important to understand the molecular mechanism of LncRNA in chondrocytes.

Treat chondrocytes with LPS to simulate the immune microenvironment of cartilage in rheumatoid arthritis *in vitro*. LPS can inhibit the proliferation of chondrocytes and promote the expression of the proinflammatory factors interleukin-23 (IL-23)and interleukin-17 (IL-17). The overexpression of MEG3 can reduce the inhibitory effect of LPS on the proliferation of chondrocytes, while the expression of the proinflammatory factors IL-23 and IL-17 is downregulated. It was also found that MEG3 can increase cell proliferation and inhibit inflammation after overexpression of MEG3 in the rat RA model (82).

## 4 The Prospect of Lncrnas in the Diagnosis and Treatment Of RA

As discussed in our review, LncRNA is an important regulator of autoimmune diseases, and the abnormal expression of certain LncRNA is related to RA. These LncRNAs, which are abnormally expressed in RA, are closely related to clinical prognosis, so that they can be used as potential molecular markers to assess the course of the disease in RA patients. Using these LncRNAs as targets, attempting to intervene, and researching possible therapeutic effects can provide new ideas for the therapy of RA.

### 4.1 LncRNAs and Clinical Diagnosis

Like most joint infections, RA can cause symptoms such as pain, fever, redness, or deformation of the joints. The difference is that the joint deformation of RA is irreversible and there is no cure for the disease. The early detection and early therapy of RA is particularly important.

As we summarized earlier in our review, LncRNA is associated with a certain course of RA disease. These disease courses include synovial hyperplasia, pannus formation, increased inflammation, cartilage damage and destruction of the bone matrix. Hence, it is speculated that LncRNA could be used as a diagnostic marker for RA to further understand the condition and formulate the best therapy plan. There is much evidence to suggest that some LncRNAs are closely related to the occurrence and development of RA. And there is much clinical data to show that some LncRNAs have apparently abnormal expression in the lesions and in the tissue prior to the lesion of RA patients. In **Table 1** we summarize the functions and mechanisms of some LncRNAs in RA.

**Table 1:** Summarization of the cellular functions of LncRNAs in RA.

LncRNAs	Expression in RA	Functions	Pathway/Target/Mechanisms	References
Lnc-IL7R	Up-regulation	Promotes cell proliferation, cell cycle progression and inhibits apoptosis of FLSs.	Interacts with the enhancer of EZH2 to promote the growth of FLSs.	(83)
HOTAIR	Down-regulation	Promote cell proliferation and inhibit inflammation of chondrocytes.	HOTAIR reduces the progression of rheumatoid arthritis by targeting miR-138 and inactivating the NF-κB pathway.	(84)
NTT	Up-regulation	Promote the differentiation of monocytes and promote the production of pro-inflammatory chemokines.	NTT is regulated by the monocyte key transcription factor C/EBPβ and that it binds to the promoter of nearby gene PBOV1 *via* hnRNP-U.	(85)
LERFS	Down-regulation	Inhibit proliferation, migration and invasion of FLSs.	LERFS specifically binds to hnRNP Q and forms a functional LERFS-hnRNP Q complex, which reduces its mRNA stability or translation by binding to the mRNA of RhoA, Rac1 and CDC42.	(86)
LINC01882	Down-regulation	Activate immune cells	LINC01882 affects the expression of transcription factor ZEB1 and kinase MAP2K4, which in turn affects the activation of T cells.	(87)
GAPLINC	Up-regulation	Promote proliferation, migration, invasion and production of pro-inflammatory cytokines of FLSs.	Increased GAPLINC expression promotes the tumor-like biological characteristics of RA-FLS	(88)
UCA1	Down-regulation	Inhibit proliferation and promote apoptosis of FLSs.	UCA1 affects the vitality of FLSs by changing the expression of Wnt6	(57)
ZFAS1	Up-regulation	Promote cell migration and invasion of FLSs	ZFAS1 promotes cell migration and invasion by interacting with miR-27a in RA-FLS	(89)
GAS5	Down-regulation	Inhibit the production of pro-inflammatory cytokines and promote apoptosis of FLSs.	GAS5 overexpression improves RA by downregulating IL-18 and inducing the apoptosis of FLSs.	(23)
DILC	Down-regulation	Inhibit the production of pro-inflammatory cytokines and promote apoptosis of FLSs.	Overexpression of lncRNA DILC may improve RA by down-regulating IL-6 and inhibiting the apoptosis of HFLS.	(90)
PICSAR	Up-regulation	Promote proliferation, migration, invasion and production of pro-inflammatory cytokines of FLSs.	PICSAR functioned through sponging miR-4701-5p in RA-FLSs.	(50)
MEG3	Down-regulation	Promote proliferation and inhibit inflammation of chondrocytes.	MEG3 inhibits RA through miR‐141 and inactivation of AKT/mTOR signalling pathway.	(82)
PVT1	Up-regulation	Promote proliferation and inflammation, inhibit cell apoptosis of FLSs.	PVT1 restores sirt6 expression by reducing sirt6 methylation, thereby reducing RA	(91)
NEAT1	Up-regulation	Promote inflammation.	NEAT1 promotes the differentiation of CD4 + T cells into Th17 cells.	(35)
HIX003209	Up-regulation	Promote the proliferation and activation of inflammatory macrophages.	HIX003209 functions as a ceRNA and exaggerates inflammation by sponging miR-6089 through TLR4/NF-κB pathway in macrophages.	(15)
LncRNA GM26870	Up-regulation	Promote the maturation of osteoclasts.	LncRNA GM26870 mediated activation of NFATC1 up-regulates the expression of CTSK, which in turn promotes the maturation of osteoclasts and aggravates the destruction of bone matrix.	(79)
MALAT1	Down-regulation	Inhibit proliferation and inflammation, promote apoptosis of FLSs.	MALAT1 affects FLSs proliferation and inflammation by promoting CTNNB1 promoter methylation and inhibiting Wnt signaling pathway	(92)
LncRNA-Jak3	Up-regulation	Promote the maturation of osteoclasts.	LncRNA-Jak3-mediated activation of NFATC1 up-regulates the expression of cathepsin K (CTSK), which in turn promotes the maturation of osteoclasts and aggravates the destruction of bone matrix.	(78)
THRIL	Up-regulation	Promote proliferation and inflammation, inhibit apoptosis of FLSs.	THRIL mediates cell growth and inflammatory response of FLSs by Activating PI3K/AKT Signals in RA.	(93)
RP11–83J16.1	Up-regulation	Promote proliferation, migration, invasion and inflammation, reduce apoptosis of FLSs.	RP11-83J16.1 activates the β-catenin pathway by regulating the URI1 to stimulate the cellular functions of RA-FLS.	(94)
HOTTIP	Up-regulation	Promote proliferation, migration, invasion and inflammation, reduce apoptosis of FLSs.	HOTTIP silencing in RA through SFRP1 promoter demethylation.	(70)
LINC01197	Down-regulation	Inhibit proliferation and inflammation, promote apoptosis of FLSs.	LINC01197 sponges miR-150 to promote THBS2 expression, leading to TLR4/NF-κB inactivation, and ameliorates RA inflammation.	(95)
IFNG-AS1	Up-regulation	Promote inflammation of peripheral blood.	Promote inflammation by catalyzing the production of IFN-γ.	(29)
H19	Up-regulation	Promote inflammation of FLSs.	DDR-2 is responsible for regulating the expression of IL-15 and Dkk-1 in RA FLS and is involved in the promotion of inflammation and joint destruction in the pathophysiological development process of RA.	(96)
FER1L4	Down-regulation	Reduce the production of inflammatory cytokines of FLSs.	FER1L4 regulates RA by targeting NLRC5.	(97)
GAS5	Down-regulation	Inhibit proliferation and inflammation of FLSs.	CAS5 regulates rheumatoid arthritis by targeting homeodomain-interacting protein kinase 2	(98)
WAKMAR2	Down-regulation	Inhibit proliferation, migration and invasion of FLSs.	WAKMAR2 acts as a competitive sponge for miR-4478, inhibits the downstream E2F1/p53 signaling pathway, and then reduces the migration and invasion characteristics of FLSs	(69)
NEAT1	Up-regulation	Promote proliferation of FLSs.	NEAT1 can activate the MAPK/ERK signaling pathway, thereby promoting the proliferation of FLS in RA	(51)
LncRNAS56464.1	Up-regulation	Promote proliferation of FLSs.	lncRNAS56464.1 affects the proliferation of FLSs in RA by targeting the miR-152-3p/Wnt pathway	(99)
MIAT	Down-regulation	Inhibit the production of inflammatory cytokines of macrophages.	LncRNA MIAT inhibits the expression of IL-1β and TNF-ɑ and affects the progress of RA	(100)
XIST	Up-regulation	Promote proliferation and inhibit apoptosis of FLS.	Down-regulating XIST can inhibit the proliferation of FLSs by increasing the expression level of miR-126-3p/NF-κB, and at the same time increase the apoptosis rate of FLSs, thereby inhibiting the occurrence and development of RA.	(61)
SNHG14	Up-regulation	Promote proliferation and inflammation of THP1.	Promotes the Jun N-terminal kinase (JNK) signaling *via* the miR-17-5p/MINK1 axis.	(101)
ZNF667-AS1	Down-regulation	Promote the proliferation of chondrocytes and inhibit inflammation in peripheral blood.	Sponging miR-523-3p and inactivating the JAK/STAT signalling.	(54)
LINC00152	Up-regulation	Promote inflammation of FLSs.	LINC00152/NF-κB feedback loop promotes RAFLS inflammation *via* regulating miR-103a/TAK1 axis and YY1 expression.	(20)

### 4.2 LncRNAs and RA Therapy

The therapy of RA is mainly divided into medical therapy and surgical therapy. Although a variety of new therapeutics and surgical methods are being used to treat RA, the prognosis has improved significantly compared to before. However, RA is still incurable and some patients would eventually become disabled. Hence, there is a great need to develop new drugs to treat RA.

#### 4.2.1 Therapeutic Drugs for RA

Drug therapy remains the main strategy of RA therapy. Current therapeutic drugs for RA are divided into four categories: non-steroidal anti-inflammatory drugs (NSAIDs), glucocorticoids (GCs), DMARDs, and biological agents (102).

NSAIDs are chemicals with anti-inflammatory and antipyretic effects (103). NSAIDs can quickly reduce inflammation and relieve pain in the therapy of RA, but have no therapeutic effect on joint destruction. And it is accompanied by side effects such as digestive tract damage (104) and liver function damage (105). Glucocorticoid enters the cytoplasm and binds to the glucocorticoid receptor (GR), then transfers it to the nucleus, where it binds to DNA and reduces the transcriptional program of pro-inflammatory cytokines (106). So as to reduce inflammation quickly and effectively. But GCs can also be accompanied by side effects such as osteoporosis, hyperglycemia, cardiovascular disease and infection (107). These side effects are also the reason why GCs can only be used for a short time and in low doses. Compared to other types of drugs, DMARDs have a slower onset of action, but can continue to alleviate the patient’s disease activity and play a better role in delaying or preventing the development of the disease (108). Nevertheless, these drugs still cannot completely cure RA. Therefore, finding new molecular targets is of great significance for improving the clinical treatment strategy of RA.

Currently, the newly developed biological agents and TNF inhibitors for the therapy of RA are milestones in the history of RA therapy, opening up a new stage in the therapy of RA with biological agents. A large number of clinical trials have shown its encouraging effects, but it still cannot alleviate the condition of all patients with RA and has obvious adverse reactions (109).

#### 4.2.2 Therapeutic Surgery of RA

Surgery is an effective treatment for RA patients with poor drug treatment and severe joint dysfunction.

There are four treatment methods for the surgical therapy of RA: Synovectomy, Arthroplasty, Arthrodesis and Corrective osteotomy (110). In the early stages of RA, joint-sparing synovectomy is used. In the early stages of RA, synovectomy can be used to preserve the joint. In the late stage of RA, joints destroy furniture and joint function can be restored by arthroplasty. Arthrodesis and corrective osteotomy should only be used when it is difficult to treat, the cartilage tissue is in poor condition, the bone quality is limited, or the patient’s general condition is poor.

However, neither medication nor surgical therapy can completely cure RA. Therefore, finding new molecular targets is of great significance for improving the clinical therapy strategy of RA.

#### 4.2.3 LncRNAs as a Target for New Drug Development

There are several mechanisms of LncRNA regulation in the incidence and development of RA. With the in-depth research on LncRNA, it has become a reality to use LncRNA as the target of new drug development, and it has a promising future in the therapy of RA.

At present, the research of new drugs targeting LncRNAs has made some progress. SiRNAs, antisense oligonucleotides (ASOs), and small molecule inhibitors have been developed. In addition, some drugs used in the clinical treatment of RA have also been found to have a therapeutic effect by targeting specific LncRNAs.

For example, siRNA targeting RA-related LncRNA (such as LOC100506036) has been shown to inhibit the inflammatory response of RA (111). Another study found that the use of siRNA to disrupt the expression of LncRNAS56464.1 from FLSs inhibited the proliferation of FLSs (52). RNAi-mediated mRNA degradation occurs in the cytoplasm (112), so siRNA can rapidly degrade LncRNAs in the cytoplasm. But RNase-H-mediated knockdown is more likely to inhibit LncRNAs in the nucleus, since RNase H mainly exists in the cell nucleus (113). For example, the use of ASO reduces the expression of LncRNA NEAT1 in peripheral T cells, thereby reducing the expression of TNF-α in the cells, which can reduce the inflammatory response of T cells (114). 5-aza-2-deoxycytidine (5-azadC), a methylation inhibitor, inhibited hypermethylation of MEG3 promoter and increase the expression level of MEG3 in FLSs. MEG3 can reduce the expression level of inflammatory cytokines in FLSs. Therefore, the use of 5-azadC methylation inhibitors in clinical trials increases the MEG3 levels in FLSs cells to exert anti-inflammatory effects (14). Shikonin is a major chemical component of Zicao, which has anti-inflammatory properties and the ability to mediate cellular and humoral immunity (115). Shikonin dose-dependently increased acetylation of histone H3 at the promoter of LncRNA NR024118. Shikonin can inhibit the secretion and expression of inflammatory cytokines such as interleukin-6 (IL-6)and interleukin-8 (IL-8) in FLSs and knockdown of LncRNA NR024118 can reverse the regulation of Shikonin on the expression of IL-6 and IL-8 in FLSs (116). In the previous article, we mentioned that LLDT-8 is a new type of triptolide derivative that shows good therapeutic effects in RA. Phase I clinical trials with this compound have been completed in rheumatoid arthritis patients (117). LLDT-8 upregulates the expression of lncRNA WAKMAR2, which is negatively correlated with the proliferation and invasion of FLSs and the production of proinflammatory cytokines. Knockout of WAKMAR2 can weaken the therapeutic effect of LLDT-8 on RA (69). These studies have shown that drugs that target LncRNAs can have broad prospects in the clinical diagnosis and treatment of RA.

### 4.3 LncRNAs and the Prognosis of RA

Many recent studies have shown that the expression levels of some molecules, including LncRNAs, in peripheral blood and in RA lesions can change significantly after drug treatment.

For example, triptolide (TPL) can downregulate the expression of LncRNA ENST00000619282, promote apoptosis and reduce joint inflammation of FLSs in RA (41). Astragaloside (AST) can reduce the expression level of LncRNA LOC100912373 in FLSs, and offset the proliferation of FLSs by LOC100912373 (52). Tanshinone IIA (Tan IIA) can up-regulate the expression of GAS5 in FLSs, reduce cell viability and promote apoptosis (118). These evidences show that the level of expression of LncRNAs may well reflect the disease state. Humans can assess the severity of disease from RA by detecting the level of expression of LncRNAs associated with RA.

With a deeper understanding of LncRNAs, more LncRNAs would be used in research into prognostic biomarkers for RA, which would provide people with more effective help in the therapy of RA.

## 5 Conclusion and Perspective

LncRNAs play an important role in the development, diagnosis and therapy of RA. At present, the mechanism of operation of some LncRNAs in the development of RA is clearly understood (**Table 1**). Some LncRNAs have been implicated in exacerbating inflammation and disrupting the immune system in RA patients. The clinical symptoms of RA patients (such as synovial hyperplasia, pannus formation, and bone damage) have also been linked to the imbalance in LncRNAs. Some LncRNAs promote the secretion of inflammatory cytokines, induce the imbalance of the immune microenvironment, induce the proliferation of FLSs and inhibit the apoptosis of FLSs, promote pannus formation, promote the proliferation and maturation of osteoclasts, and exacerbate bone damage, thereby promoting the development of RA. However, some LncRNAs have also been shown to protect the immune system and joints by inhibiting the secretion of inflammatory factors, maintaining the balance of the immune system, inhibiting the cellular activity of FLSs, promoting cell apoptosis, and inhibiting the activity of osteoclasts, thereby slowing the onset of RA. Therefore, LncRNAs show promise to serve as important therapeutic targets for RA.

However, the potential molecular mechanisms of LncRNAs in RA are still largely unknown. In the future, larger studies are needed to increase our understanding of the changes of LncRNAs and the development of RA. This will shed new light on the development of potential drug targets for RA.

In short, LncRNAs provide new ideas for the clinical diagnosis and therapy of RA.

## Author Contributions

WH and XL: conceptualization and compilation of data. WH, XL, and WC: writing part and proofreading. WH, XL, and QZ: designing of figure. CH and YT: Collection of relevant references. WH, XL, QZ, and WC: language polishing. All authors contributed to the article and approved the submitted version.

## Funding

This work was supported by the National Natural Science Foundation of China (81471659), Guangzhou Science and Technology Planning Project (202103000002), Guangdong Medical Science and Technology Research Foundation (B2021376) and Panyu Major Science and Technology Planning Project (2020-Z04-002).

## Conflict of Interest

The authors declare that the research was conducted in the absence of any commercial or financial relationships that could be construed as a potential conflict of interest.

## Publisher’s Note

All claims expressed in this article are solely those of the authors and do not necessarily represent those of their affiliated organizations, or those of the publisher, the editors and the reviewers. Any product that may be evaluated in this article, or claim that may be made by its manufacturer, is not guaranteed or endorsed by the publisher.
